# An explainable fairness-aware AI framework for exam score verification

**DOI:** 10.3389/frai.2026.1824849

**Published:** 2026-07-02

**Authors:** Julius Olaniyan, Silas Formunyuy Verkijika, Ibidun C. Obagbuwa

**Affiliations:** 1 Center for Applied Data Science (CADS), Faculty of Natural and Applied Sciences, Sol Plaatje University, Kimberley, South Africa; 2 Department of Computer Science & Information Technology, Faculty of Natural and Applied Sciences, Sol Plaatje University, Kimberley, South Africa; 3 Department of Mathematical Science and Computing, Walter Sisulu University, Mthatha, South Africa

**Keywords:** automated essay scoring, explainable artificial intelligence, fairness-aware AI, peer-consistency analysis, rubric alignment

## Abstract

Ensuring fairness and reliabilityin examination scoring remains a persistent challenge in educational assessment, particularly in the presence of subjective grading inconsistencies across evaluators. While automated essay scoring systems improve scalability, limited attention has been given to mechanisms for verifying whether assigned scores are consistent with rubric criteria and comparable peer responses. This study proposes an Explainable Fairness-Aware AI Framework for Exam Score Verification, designed to detect potential scoring anomalies while providing interpretable evidence to support verification decisions. The proposed framework integrates three complementary components: semantic response evaluation using contextual representations derived from DeBERTa-v3 embeddings, criterion-level rubric alignment via cross-attention mechanisms, and peer-consistency analysis based on similarity-driven cohort score distributions. To enhance transparency, an explainability layer combines attention visualization, integrated gradients, and natural language justification to provide both quantitative and qualitative insights into model decisions. The framework was evaluated on the ASAP 2.0 dataset comprising 24,728 rubric-scored essays. Experimental results, reported as mean ± standard deviation over multiple runs, demonstrate that the proposed approach achieves an agreement of 87.1% ± 0.9 with peer-consistent reference scores and a fairness consistency index of 0.82 ± 0.02, outperforming a diverse set of baseline models, including traditional, transformer-based, and hybrid scoring approaches. Ablation studies further confirm the critical role of peer-consistency analysis in detecting scoring irregularities, while the explainability components enhance interpretability without significantly affecting predictive performance. The notion of fairness addressed in this work is grounded in score consistency relative to rubric expectations and semantically similar peer responses. Within this scope, the results indicate that integrating semantic evaluation, rubric-aware modeling, and cohort-referenced analysis provides a robust and interpretable framework for exam score verification. These findings highlight the potential of multi-evidence, explainable AI systems to support reliable and transparent assessment in educational settings.

## 1 Introduction

The use of artificial intelligence in educational assessment has expanded rapidly as institutions seek scalable solutions for evaluating student performance (Alsharif, 2025). Automated scoring systems, predictive assessment models, and AI-assisted moderation platforms are now increasingly integrated into digital learning environments to manage large examination cohorts and accelerate grading workflows (Mahamad et al., 2025). These technologies promise improvements in efficiency and consistency; however, their growing influence in academic decision-making raises critical concerns regarding algorithmic fairness, transparency, and institutional accountability (Cheong, 2024). Events surrounding algorithm-driven grading during the disruption caused by the COVID-19 highlighted the risks of opaque computational systems in high-stakes evaluation contexts, where insufficient transparency and limited oversight can undermine trust in assessment outcomes (Kwakye, 2024).

Within formal examination systems, score verification serves as an essential mechanism for maintaining fairness and procedural integrity (Aloisi, 2023). Conventional verification processes rely primarily on manual rechecking and administrative review, which are often slow, resource-intensive, and prone to inconsistencies across large student populations (Jayaram and Bhat, 2022). As AI-supported grading becomes more prevalent, these traditional verification procedures are increasingly inadequate (Deepshikha, 2026). Instead, there is a growing need for intelligent verification mechanisms capable of systematically auditing algorithmic decisions, detecting irregularities in predicted scores, and providing interpretable explanations for those outcomes (Nangi et al., 2022). Such systems must balance three critical requirements: statistical robustness in detecting anomalies, equitable treatment across diverse demographic and linguistic groups, and transparent explanations that stakeholders can meaningfully interpret (Ayeola, 2025).

Despite the rapid advancement of automated assessment technologies, much of the existing research concentrates on improving prediction accuracy in automated scoring tasks, including essay evaluation, short-answer grading, and objective test assessment (Ramesh and Sanampudi, 2022). Comparatively little attention has been directed toward the verification stage that follows automated scoring (Lin et al., 2024). In many implementations, fairness considerations are introduced only after models have been deployed, typically through *post-hoc* adjustments or bias mitigation strategies (Moon and Ahn, 2025). This reactive design paradigm is particularly problematic in educational environments characterized by linguistic diversity, cultural variation, and unequal access to learning resources (Akintayo et al., 2024). Under such conditions, AI systems may unintentionally replicate or intensify pre-existing disparities if fairness safeguards are not embedded into the assessment pipeline from the outset.

To address these limitations, this study proposes an Explainable Fairness-Aware AI Framework for Exam Score Verification (EFAI-ESV). The framework is designed to function as an auditing layer that systematically examines AI-generated examination scores before they are finalized or released. It combines three complementary components. First, a fairness-aware anomaly detection module identifies unusual scoring patterns and potential disparities across relevant subgroups. Second, an interpretable auditing mechanism applies feature attribution and counterfactual reasoning techniques to explain why certain scores are flagged for verification. Third, a recalibration mechanism adjusts predictions when bias or inconsistencies are detected, thereby preserving both fairness and predictive reliability. By integrating these components, the framework embeds transparency and equity considerations directly into the verification stage rather than treating them as post-processing corrections.

From a methodological perspective, the proposed framework employs distributional consistency analysis, subgroup calibration evaluation, and causal fairness metrics to determine whether predicted examination scores align with expected academic performance patterns. The explainability layer further generates structured rationales for flagged cases, enabling educators and administrators to understand the basis of algorithmic verification decisions. Importantly, the framework is designed to operate independently of specific grading models, allowing it to be integrated with a wide range of automated assessment systems, including objective examinations, essay-based evaluations, and hybrid multimodal assessments.

By reframing score verification as an algorithmic auditing problem, this work contributes a governance-oriented perspective to the development of AI-enabled educational technologies. Rather than focusing solely on improving predictive performance, the proposed approach emphasizes accountability and fairness as foundational properties of automated assessment systems. Establishing transparent and fairness-aware verification mechanisms is essential if AI-driven grading systems are to gain long-term credibility within educational institutions. Consequently, this research seeks to advance the discussion beyond the technical feasibility of automated grading toward a more fundamental question: whether AI-based assessment systems can provide decisions that are not only accurate, but also explainable, equitable, and defensible within academic governance structures.

## 2 Related work

### 2.1 AI-Based automated assessment systems

AI-driven automated grading systems have become increasingly prominent in higher education to handle large volumes of student submissions efficiently. Dimari et al. (2024) explored automated grading for open-book examinations and demonstrated that AI can maintain scoring consistency while reducing evaluation time. Similarly, Yashas et al. (2025) developed an AI-driven assessment system integrating natural language processing and machine learning to generate automated scores and rapid feedback. These studies highlight the potential of AI to streamline assessment processes.

Further evidence of the viability of automated scoring systems is provided by Seneviratne and Manathunga (2025) who demonstrated that AI-assisted short-answer scoring tools exhibit strong correlations with human examiner scores. However, AI-based scoring systems often face limitations in handling ambiguous or context-dependent responses. Ercikan and McCaffrey (2022) emphasized the need for evidence-centered design frameworks to ensure that AI-based scoring aligns with the pedagogical objectives and constructs being measured. This indicates that while automated grading can improve efficiency, its effectiveness depends on careful design to preserve assessment validity.

### 2.2 Fairness in AI-based educational assessment

Concerns about fairness have become central in educational AI systems. Machine learning models can inadvertently reproduce historical biases in student data, leading to inequitable outcomes. Hanifa (2025) analyzed algorithmic bias in AI-supported assessment and personalized learning, stressing the importance of equity principles. While these studies provide frameworks for detecting bias, they often focus solely on predictive accuracy without integrating corrective mechanisms.

Similarly, Kesgin et al. (2025) and collaborators explored fairness-aware machine learning techniques for predicting student academic performance. Addressing fairness concerns in educational AI systems also requires mechanisms that account for linguistic and contextual diversity among learners. In this context, Kumar et al. (2025) proposed an adaptive AI-based assessment system that integrates explainability mechanisms with human-in-the-loop bias correction.

Human–AI collaborative systems have emerged as a complementary approach to address fairness concerns. Zeng et al. (2024) and Xiao et al. (2025) proposed hybrid essay scoring frameworks where AI generates initial evaluations and humans review or adjust scores, combining efficiency with fairness oversight. Such approaches demonstrate that incorporating human expertise can mitigate biases inherent in fully automated systems.

### 2.3 Alternative assessment approaches: peer assessment

Peer assessment offers a non-AI method to improve scalability and feedback while maintaining validity. El Alaoui (2023) proposed a fuzzy coherence measure to improve peer assessment reliability, while Sofjan et al. (2023) validated the use of peer scores and feedback in online learning platforms. Gumala (2025) explored peer assessment's impact on final exam performance, highlighting benefits for learner engagement and perspective diversity. While effective, peer-based methods are labor-intensive and less consistent than automated approaches, suggesting that hybrid AI-human solutions may offer the best balance.

### 2.4 Explainable and trustworthy AI for educational decision systems

In high-stakes educational assessments, explainability is essential to ensure transparency, trust, and accountability. Basaran (2026) showed that large language models for automated and human scoring become more interpretable when stakeholders can trace score generation, which also facilitates bias detection. Expanding on this, Akter et al. (2025) combined causal and predictive analysis with XAI to evaluate socio-academic and economic factors affecting student performance, demonstrating that transparency can reveal not only scores but also the contextual influences on learning outcomes.

Recent work has focused on embedding explainability directly into essay scoring. Xu et al. (2025) introduced rubric-aligned chain-of-thought prompting, aligning AI-generated evaluations with human grading standards while preserving interpretability. Najjar et al. (2025) leveraged XAI to detect AI-generated student text, supporting academic integrity and demonstrating the ethical potential of transparent AI in assessment.

Complementary research highlights the synergy between explainability and fairness. Matta and Bolli (2023) argued that interpretability can enhance trust by making predictions understandable, while Gouda (2025) proposed a framework that identifies hidden biases in educational scoring systems through interpretable machine learning techniques. Markova (2025) further emphasized governance by integrating fairness detection and bias mitigation into regulation-aware AI frameworks.

Despite these advances, most studies treat explainability as an isolated objective rather than combining it with fairness-aware scoring, bias mitigation, or auditing in operational educational systems. This gap underscores the need for integrated frameworks that ensure AI assessments are interpretable, equitable, and accountable precisely the focus of the present study.

### 2.5 AI Auditing and fairness certification

Auditing and certification frameworks provide systematic evaluation of fairness and compliance in AI systems. Agarwal et al. (2023) proposed a fairness score framework to standardize evaluation procedures, while Prakash et al. (2026) introduced the Nishpaksh framework for TEC-compliant fairness auditing. Landers and Behrend (2023) emphasized evaluating both models and the processes used to generate predictions, highlighting the need for system-level transparency. These frameworks are important, but they rarely focus on individual scoring instances in educational assessments, leaving a gap for frameworks that combine auditing with score verification.

While prior research has advanced automated grading, fairness-aware machine learning, XAI, human-AI hybrid systems, and auditing frameworks, these areas are typically investigated independently. Few studies integrate automated score verification, bias detection, explainable reporting, and hybrid oversight within a single framework. This fragmentation limits trust and reliability in high-stakes assessment environments. To address these gaps, this study proposes an Explainable Fairness-Aware AI Framework for Exam Score Verification. By integrating automated grading, fairness-aware anomaly detection, human–AI hybrid validation, and interpretable auditing, the framework ensures transparent, equitable, and reliable assessment outcomes, bridging the isolated approaches highlighted in prior literature.

## 3 Methodology

This section described the proposed Explainable Fairness-Aware AI Framework for exam score verification. The methodology was designed to address three key limitations of existing assessment-support systems: (i) the lack of systematic mechanisms for verifying the fairness and consistency of lecturer-assigned scores, (ii) limited integration of rubric- and cohort-level contextual information when analyzing assessment outcomes, and (iii) insufficient explainability to support transparent score dispute resolution. To address these challenges, the framework employed a DeBERTa-v3 transformer as the semantic backbone, due to its disentangled attention mechanism and relative position encoding, which provided superior semantic fidelity and long-range dependency modeling critical for comparing conceptually similar student responses exhibiting substantial lexical and stylistic variation. This semantic module was integrated with rubric-aware cross-attention modeling and similarity-conditioned cohort score consistency analysis, forming a multi-evidence verification architecture designed to provide interpretable, fairness-aware audit signals within a human-in-the-loop framework.

### 3.1 Problem formulation

The exam score verification task was formulated as a fairness auditing problem, where the objective was to determine whether a lecturer-assigned score for a student essay was consistent with semantic quality, rubric criteria, and peer response consistency. Unlike conventional automated essay scoring, the framework did not aim to predict the score, but to verify its fairness relative to multiple evidence signals as describe in Equation 1.

Formally, let the dataset be denoted as:


$$D=\left({\text{x}}_{\text{i}},{\text{r}}_{\text{i}},{\text{s}}_{\text{i}},{\text{p}}_{\text{i}}\right){\}}_{\text{i}=1}^{\text{N}}$$ (1)

where:

*x*_*i*_ represents the textual content of the *i*-th student essay from the ASAP 2.0 dataset,

*r*_*i*_ represents the associated rubric criteria for the essay's prompt,

*s*_*i*_∈[0, *S*_max_] is the human-assigned holistic score, and

*p*_*i*_ denotes the prompt identifier corresponding to the essay's writing task.

Each essay in ASAP 2.0 was accompanied by prompt-specific rubric constraints, source passages (for evidence-based prompts), and metadata fields. Only the textual content and rubric information were used as inputs for verification modeling, while demographic metadata (e.g., race_ethnicity, gender, economically_disadvantaged) was excluded from predictive calculations and reserved solely for *post hoc* fairness analysis.

#### 3.1.1 Inputs and outputs

The inputs to the framework included:

1. Student essay text (**x**_**i**_).
2. Prompt-specific rubric (**r**_**i**_).
3. Peer essay subset (P_**i**_), defined as other essays responding to the same prompt with high semantic similarity (*Sim***(x**_**i**_**, x**_**j**_**)≥ τ**).

The output was a fairness verification label and associated confidence as seen in Equation 2:


$$\begin{array}{l} f:\left(x_{i},r_{i},P_{i}\right)\to \left({\text{ŷ}}_{i},c_{i}\right) \end{array}$$ (2)

where:

ŷ_*i*_∈{*fair, under*−*scored, over*−*scored*}

*c*_*i*_∈[0, 1] represented the confidence score in the fairness assessment.

#### 3.1.2 Peer score definition

Given the absence of multiple human scores per essay in ASAP 2.0, peer scores were defined in Equation 3 as the scores assigned to other essays in the same prompt exhibiting high semantic similarity to the target essay:


$$\begin{array}{l} {\text{P}}_{\text{i}}=\left\{\left({\text{x}}_{\text{j}},{\text{s}}_{\text{j}}\right)|{\text{p}}_{\text{j}}={\text{p}}_{\text{i}}\;\wedge \text{ Sim}\left({\text{x}}_{\text{i}},{\text{x}}_{\text{j}}\right)\geq \tau ,\text{ j}\neq \text{i}\right\} \end{array}$$ (3)

The peer score distribution was used to evaluate whether the target score *s*_*i*_ was consistent with comparable essays. This approach reflected cohort-referenced moderation, a standard practice in educational assessment.

#### 3.1.3 Assumptions

The problem formulation relied on the following assumptions:

1. Rubric fidelity: the prompt-specific rubric reflected intended learning outcomes and provided a reliable criterion for evaluation.
2. Semantic comparability: essays with high semantic similarity were expected to receive similar scores under the rubric.
3. Score consistency: deviations from peer scores were meaningful indicators of potential unfairness rather than inherent errors in peer scoring.
4. Demographic neutrality: demographic attributes did not influence predictions; the framework focused on content and rubric alignment.

### 3.2 Framework overview

**Figure 1:**
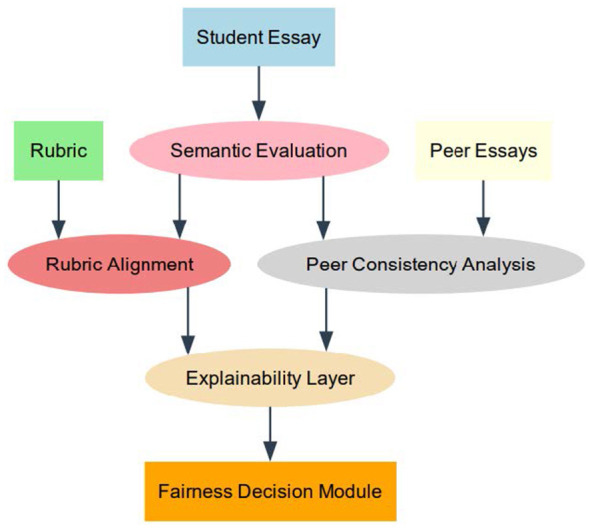
Architectural overview of the proposed framework.

The proposed Explainable Fairness-Aware AI Framework operated as a multi-evidence system designed to verify the fairness of lecturer-assigned exam scores. As illustrated in Figure 1, the framework ingested a student essay, its corresponding rubric, and a set of peer essays identified through semantic similarity. The student essay was first processed by a DeBERTa-v3 semantic encoder to generate contextual embeddings capturing conceptual meaning, which were subsequently leveraged by a rubric alignment module employing cross-attention to quantify criterion-level fulfillment. Simultaneously, the semantic embeddings were used to analyze the cohort-based peer score distribution, producing a statistical measure of consistency relative to conceptually similar responses. Outputs from these modules were integrated within an explainability layer, which provided attention visualizations, feature attributions, and natural language justifications. Finally, a fairness decision module fused the semantic, rubric, and peer evidence to generate a verification label such as fair, under-scored, or over-scored alongside a confidence score. This modular design enabled parallel extraction of independent fairness signals while maintaining centralized, interpretable decision fusion, ensuring that each verification outcome was both evidence-based and explainable.

### 3.3 Dataset description

The experiments were conducted using the ASAP 2.0 Automated Student Assessment Prize dataset, which is publicly available on Kaggle (Burleigh, 2024), and has been widely adopted in automated essay scoring research due to its scale, diversity, and the availability of prompt-specific rubric constraints. This dataset comprised a total of 24,728 student essays spanning multiple writing prompts, each graded by human raters according to explicit rubric criteria, thereby providing a reliable benchmark for evaluating automated assessment systems. Each essay instance included 14 attributes capturing the textual content, task context, and demographic metadata. The full_text field contained the complete student-written essay, while the score variable represented the human-assigned holistic score, serving as the primary supervision signal for analysis. Task context was provided through fields such as assignment and prompt_name, specifying the writing task and associated prompt category. For evidence-based prompts, up to four source passages were included (source_text_1 through source_text_4), enabling modeling of content relevance and evidence usage. While demographic attributes such as economically disadvantaged status, disability status, English language learner status, race, ethnicity, and gender were included, these variables were excluded from the predictive pathways to avoid introducing bias, but they were considered during *post hoc* fairness-oriented analysis. The dataset structure facilitated both semantic evaluation of essay content and rubric-driven assessment, making it well-suited for the proposed multi-evidence verification framework.

While the ASAP 2.0 dataset provides only a single human-assigned score per essay, we simulate a second human marker using a peer-consistency-based reference score. Specifically, each essay's score is compared against the mean score of semantically similar essays within the same prompt group, forming a proxy evaluation that approximates independent human judgment. This approach enables measurement of agreement with a “second marker” and supports fairness-aware verification despite the single-annotator limitation.

To minimize validation bias associated with peer-referenced proxy markers, the construction of the proxy score is performed strictly using embedding-based semantic similarity derived from the essay text, and is independent of the human-assigned score of the target essay. Specifically, the target essay is excluded from the computation of its own proxy reference, and peer selection is based solely on textual similarity within the same prompt group. This ensures that the proxy marker does not directly or indirectly incorporate the ground-truth score of the evaluated instance, thereby reducing circular dependency in the evaluation process. Furthermore, the proxy score is treated as a relative consistency signal rather than an absolute ground-truth replacement, allowing it to capture cohort-level agreement patterns without reinforcing individual scoring biases present in the dataset. This design reduces evaluation bias by decoupling score supervision from cohort formation, ensuring that agreement metrics reflect semantic consistency rather than score-induced clustering.

### 3.4 Data preprocessing

Prior to model training and evaluation, a comprehensive preprocessing pipeline was applied to the dataset to ensure consistency and maximize the quality of feature representations. The textual content of each student essay was first subjected to normalization, which included converting all text to lowercase, removing extraneous whitespace, and standardizing punctuation and special characters. Tokenization was then performed using the DeBERTa-v3 tokenizer, which ensured alignment with the transformer's pre-trained embedding vocabulary and preserved subword representations for out-of-vocabulary terms. Stopwords were retained to maintain contextual information critical for semantic comprehension, and sentences were segmented to support potential downstream attention-based analysis. The rubric information, including assignment prompts and scoring criteria, was structured and encoded to form vector representations suitable for cross-attention modeling. Source passages associated with evidence-based prompts were similarly encoded, allowing the model to evaluate essay content relative to expected references. Peer essays were also tokenized and encoded consistently to enable accurate computation of semantic similarity metrics for cohort-based fairness analysis. This preprocessing pipeline ensured that all textual inputs, whether student essays, rubrics, or peer essays, were transformed into high-fidelity embeddings capable of supporting both semantic evaluation and rubric alignment in the proposed verification framework.

### 3.5 Semantic answer evaluation module

The Semantic Answer Evaluation Module constituted the foundational component of the proposed framework, responsible for encoding the conceptual meaning of student essays and supporting subsequent rubric and peer-based fairness analysis. Each student essay *x*_*i*_ was processed using the DeBERTa-v3 transformer, selected for its disentangled attention mechanism and relative position encoding, which allowed the model to capture both local contextual dependencies and long-range semantic relationships within the essay. Formally, the transformer produced a sequence of contextualized embeddings $H_{i}\in {\mathbb{R}}^{T\times d}$, where *T*represents the number of tokens in the essay and *d*denotes the embedding dimensionality as shown in Equation 4. Each token embedding *h*_*i, t*_ captured both content and positional information through the transformer's multi-head self-attention operation, defined as:


$$\begin{array}{l} {\text{h}}_{\text{i},\text{t}}^{\left(\text{l}+1\right)}=\text{LayerNorm}\\ \left({\text{h}}_{\text{i},\text{t}}^{\left(\text{l}\right)}+\overset{\text{H}}{\underset{\text{k}=1}{\sum }}\text{Attentio}{\text{n}}_{\text{k}}\left({\text{Q}}_{\text{k}}^{\left(\text{l}\right)}{\text{h}}_{\text{i}}^{\left(\text{l}\right)},{\text{K}}_{\text{k}}^{\left(\text{l}\right)}{\text{h}}_{\text{i}}^{\left(\text{l}\right)},{\text{V}}_{\text{k}}^{\left(\text{l}\right)}{\text{h}}_{\text{i}}^{\left(\text{l}\right)}\right)\right) \end{array}$$ (4)

where *H* is the number of attention heads, and $Q_{k}^{\left(l\right)},K_{k}^{\left(l\right)},V_{k}^{\left(l\right)}$ are the query, key, and value projection matrices for the *k*-th head at layer *l*. The resulting embeddings preserved fine-grained semantic distinctions while accounting for positional context, which is crucial for distinguishing essays that express the same ideas using varied syntactic structures.

Following embedding generation, semantic similarity between essays was computed to support peer-based fairness analysis and content coverage evaluation. Given a target essay embedding *H*_*i*_ and a peer essay embedding *H*_*j*_ as shown in Equation 5, the cosine similarity was calculated as:


$$\begin{array}{l} \text{Sim}\left({\text{H}}_{\text{i}},{\text{H}}_{\text{j}}\right)=\frac{1}{{\text{T}}_{\text{i}}{\text{T}}_{\text{j}}}\overset{{\text{T}}_{\text{i}}}{\underset{\text{t}=1}{\sum }}\overset{{\text{T}}_{\text{j}}}{\underset{\text{u}=1}{\sum }}\frac{{\text{h}}_{\text{i},\text{t}}^{⊺}\;{\text{h}}_{\text{j},\text{u}}}{||{\text{h}}_{\text{i},\text{t}}{||}_{2}||{\text{h}}_{\text{j},\text{u}}{||}_{2}} \end{array}$$ (5)

In addition to similarity, coverage modeling was introduced to quantify the degree to which the essay addressed prompt-specific rubric criteria. Let $R_{i}\in {\mathbb{R}}^{C\times d}$ represent the embeddings of the rubric criteria for the corresponding prompt, where *C*denotes the number of rubric dimensions as depicted in Equations 6, 7. Coverage scores were computed using a cross-attention mechanism between essay embeddings and rubric embeddings:


$$\begin{array}{l} {\text{α}}_{\text{t},\text{c}}=\frac{\exp\left({\text{h}}_{\text{i},\text{t}}\cdot {\text{r}}_{\text{i},\text{c}}\right)}{\sum_{{\text{c}}^{\text{′}}=1}^{\text{C}}\exp\left({\text{h}}_{\text{i},\text{t}}\cdot {\text{r}}_{\text{i},{\text{c}}^{\text{′}}}\right)},{\text{v}}_{\text{c}}=\overset{\text{T}}{\underset{\text{t}=1}{\sum }}{\text{α}}_{\text{t},\text{c}}{\text{h}}_{\text{i},\text{t}} \end{array}$$ (6)

$$\begin{array}{l} \text{CoverageScor}{\text{e}}_{\text{c}}=\frac{∥{\text{v}}_{\text{c}}{∥}_{2}}{\sum_{{\text{c}}^{\text{′}}=1}^{\text{C}}∥{\text{v}}_{{\text{c}}^{\text{′}}}} \end{array}$$ (7)

Here, α_*t, c*_ denotes the attention weight of token *t* relative to rubric criterion *c*, and *v*_*c*_ represents the aggregated representation of essay content aligned to that criterion. The coverage score *CoverageScore*_*c*_ quantified how well the essay addressed each rubric dimension relative to the overall content, providing a principled, criterion-level measure that informed both the rubric alignment and fairness decision modules.

By combining transformer-based embeddings, cosine similarity for peer comparison, and rubric-based coverage modeling, the Semantic Answer Evaluation Module generated rich, multidimensional representations of each essay. These representations were critical for detecting discrepancies between lecturer-assigned scores and the semantic quality of the essay, enabling the downstream modules to perform fairness-aware verification in a manner that was both quantitative and interpretable.

### 3.6 Rubric alignment scoring

The Rubric Alignment Scoring module quantified how effectively a student essay addressed the specific criteria outlined in the prompt rubric, providing a structured evaluation of criterion-level performance to support fairness verification. This module built directly upon the essay embeddings generated by the Semantic Answer Evaluation Module and the rubric embeddings derived from prompt criteria, allowing cross-modal alignment between student content and expected learning outcomes. As seen in Equations 8, 9, let $H_{i}\in {\mathbb{R}}^{T\times d}$ denote the token-level embeddings of essay *i*, and let $R_{i}\in {\mathbb{R}}^{C\times d}$ denote the embeddings of the *C* rubric criteria associated with that essay's prompt. A cross-attention mechanism was employed to compute the alignment between essay content and rubric criteria:


$$\begin{array}{l} \beta_{\text{t},\text{c}}=\frac{\exp\left({\text{h}}_{\text{i},\text{t}}\cdot {\text{r}}_{\text{i},\text{c}}\right)}{\sum_{{\text{c}}^{\text{′}}=1}^{\text{C}}\exp\left({\text{h}}_{\text{i},\text{t}}\cdot {\text{r}}_{\text{i},{\text{c}}^{\text{′}}}\right)} \end{array}$$ (8)

$$\begin{array}{l} {\text{u}}_{\text{c}}=\overset{\text{T}}{\underset{\text{t}=1}{\sum }}\beta_{\text{t},\text{c}}{\text{h}}_{\text{i},\text{t}} \end{array}$$ (9)

Here, β_*t, c*_ represents the attention weight of token *t* with respect to rubric criterion *c*, and *u*_*c*_ is the aggregated essay representation corresponding to that criterion. This approach allowed the model to capture fine-grained interactions between essay content and rubric dimensions, distinguishing cases where an essay partially addressed multiple criteria vs. fully addressing specific ones.

A criterion-level alignment score was then computed by measuring the cosine similarity between the aggregated essay representation *u*_*c*_ and the rubric embedding *r*_*i, c*_ as described in Equation 10:


$$\begin{array}{l} {\text{s}}_{\text{i},\text{c}}^{\text{rubric}}=\frac{{\text{u}}_{\text{c}}\cdot {\text{r}}_{\text{i},\text{c}}}{∥{\text{u}}_{\text{c}}{∥}_{2}∥{\text{r}}_{\text{i},\text{c}}{∥}_{2}},\text{c}=1,\ldots ,\text{C} \end{array}$$ (10)

These scores, $s_{i,c}^{rubric}$, provided a normalized measure of alignment for each rubric criterion, capturing how well the essay satisfied the specific expectations of the prompt.

To produce a holistic rubric alignment score, a weighted aggregation across criteria was performed. Let *w*_*c*_ denote the importance weight of rubric criterion *c*, which could be derived from prompt-specific scoring guidelines or uniform if no explicit weighting was provided. The aggregated rubric alignment score was computed in Equation 11 as:


$$\begin{array}{l} {\text{S}}_{\text{i}}^{\text{rubric}}=\overset{\text{C}}{\underset{\text{c}=1}{\sum }}{\text{w}}_{\text{c}}{\text{s}}_{\text{i},\text{c}}^{\text{rubric}},\overset{\text{C}}{\underset{\text{c}=1}{\sum }}{\text{w}}_{\text{c}}=1 \end{array}$$ (11)

This score summarized criterion-level alignment into a single metric reflecting the overall fulfillment of rubric expectations. Importantly, the weighted aggregation allowed the framework to prioritize key rubric dimensions when computing fairness signals, providing flexibility for prompts with unevenly emphasized criteria.

The criterion-level and aggregated rubric scores were subsequently integrated with outputs from the peer consistency module and the semantic similarity module to inform the fairness decision module in Equation 12. Specifically, the aggregated rubric score contributed to the overall fairness confidence score ĉ_*i*_, defined as:


$$\begin{array}{l} {\text{ĉ}}_{\text{i}}=\text{σ}\left(\lambda_{1}{\text{S}}_{\text{i}}^{\text{rubric}}+\lambda_{2}{\text{S}}_{\text{i}}^{\text{peer}}+\lambda_{3}{\text{S}}_{\text{i}}^{\text{semantic}}\right) \end{array}$$ (12)

where $S_{i}^{peer}$ denotes the normalized peer consistency metric, $S_{i}^{semantic}$ represents the essay's semantic coverage score, λ_1_, λ_2_, λ_3_ are learnable or empirically determined fusion weights, and σ is the sigmoid function mapping the combined evidence to the interval [0,1]. This formulation enabled the model to quantitatively integrate rubric alignment with peer and semantic signals, producing a principled, interpretable measure of potential under-scoring or over-scoring, which was then thresholded to assign the final verification label.

Leveraging token-level cross-attention, criterion-level cosine similarity, and weighted aggregation, makes the Rubric Alignment Scoring module provides a robust and interpretable measure of how closely an essay adhered to its intended learning objectives, forming a central component of the evidence-based fairness verification pipeline.

### 3.7 Peer-based fairness analysis

The Peer-Based Fairness Analysis module was designed to detect potential under-scoring or over-scoring of student essays by leveraging the distribution of scores among conceptually similar peers. Unlike traditional holistic assessment, this module incorporated cohort-referenced evaluation, which enabled the system to identify statistical inconsistencies in lecturer-assigned scores relative to peer essays responding to the same prompt in Equation 13. Let P_*i*_ denote the set of peer essays for the *i*-th student essay, selected based on semantic similarity computed from the DeBERTa-v3 embeddings *H*_*i*_ and *H*_*j*_ as previously defined:


$$\begin{array}{l} \text{Sim}\left({\text{H}}_{\text{i}},{\text{H}}_{\text{j}}\right)=\frac{1}{{\text{T}}_{\text{i}}{\text{T}}_{\text{j}}}\overset{{\text{T}}_{\text{i}}}{\underset{\text{t}=1}{\sum }}\overset{{\text{T}}_{\text{j}}}{\underset{\text{u}=1}{\sum }}\frac{{\text{h}}_{\text{i},\text{t}}\cdot {\text{h}}_{\text{j},\text{u}}}{∥{\text{h}}_{\text{i},\text{t}}{∥}_{2}∥{\text{h}}_{\text{j},\text{u}}{∥}_{2}},\text{j}\in {\text{P}}_{\text{i}} \end{array}$$ (13)

Essays exceeding a similarity threshold τ were included in the peer group in Equation 14 as:


$$\begin{array}{l} G_{i}=\left\{\text{j}|\text{Sim}\left({\text{H}}_{\text{i}},{\text{H}}_{\text{j}}\right)\geq \text{τ},\text{j}\neq \text{i}\right\} \end{array}$$ (14)

This similarity-based grouping ensured that only semantically comparable essays were used to evaluate fairness, mitigating the influence of outliers or essays with different topic coverage. To mitigate potential validation bias introduced by peer-referenced scoring, the similarity-based cohort construction was performed independently of the human-assigned scores *y*_*i*_, relying solely on embedding-derived semantic representations. This separation ensures that score information does not influence cohort formation, thereby reducing circularity in evaluation and preserving the independence of the fairness assessment pipeline.

Within each peer group G_*i*_, the distribution of human-assigned scores *y*_*j*_ was modeled as a univariate Gaussian in Equation 15:


$$\begin{array}{l} {\text{y}}_{j}\sim N\left(\mu_{i},\sigma_{i}^{2}\right),\text{j}\in G_{i} \end{array}$$ (15)

where $\mu_{i}=\frac{1}{|G_{i}|}\underset{j\in G_{i}}{\sum }y_{j}$ is the mean peer score, and $\sigma_{i}^{2}=\frac{1}{|G_{i}|}\underset{j\in G_{i}}{\sum }{\left(y_{j}-\mu_{i}\right)}^{2}$ represents the score variance within the group. The Gaussian assumption provided a principled statistical baseline for detecting discrepancies between a student's assigned score *y*_*i*_ and the peer distribution.

To ensure the stability of the Gaussian approximation, a minimum cohort size requirement was enforced. Only peer groups with a sufficiently large number of essays were included in the statistical modeling process. This ensures that the estimates of the group mean score and score variance are reliable and not distorted by small sample effects. Very small peer cohorts may produce unstable variance estimates and can violate the assumptions required for valid *z*-score normalization. Therefore, cohorts that do not meet the minimum size requirement were excluded from Gaussian-based fairness analysis to maintain statistical robustness.

Statistical inconsistency detection was performed by computing the standardized *z*-score of the target essay's assigned score relative to its peer group as seen in Equation 16:


$$\begin{array}{l} {\text{z}}_{\text{i}}=\frac{{\text{y}}_{\text{i}}-{\text{μ}}_{\text{i}}}{{\text{σ}}_{\text{i}}+\text{ϵ}} \end{array}$$ (16)

where ϵ is a small constant added to avoid division by zero. The magnitude of *z*_*i*_ indicated the degree of deviation from the peer-consistent norm. Thresholds δ_1_ and δ_2_ were applied to categorize potential under-scoring or over-scoring in Equation 17:


$${\text{Verification}}_{\text{peer}}\left(\text{i}\right)=\{\begin{array}{cc} under-scored, & z_{\text{i}}<\text{−}\delta_{\text{1}}\\ \text{over}-\text{scored}, & z_{\text{i}}>\delta_{\text{2}}\\ fair, & \text{−}\delta_{\text{1}}\leq z_{\text{i}}\leq \delta_{\text{2}} \end{array}$$ (17)

This statistical procedure provided quantitative, evidence-based indications of score fairness relative to a semantically matched cohort. By incorporating both similarity-based peer grouping and *z*-score deviation analysis, the module enabled the detection of anomalies that could suggest either inadvertent under-assessment or grading leniency.

Finally, the outputs of the Peer-Based Fairness Analysis were integrated with rubric alignment scores and semantic coverage metrics in the Fairness Decision Module. The resulting framework combined individual essay evaluation, cohort-referenced assessment, and criterion-level rubric alignment to produce robust, interpretable fairness verification labels, ensuring that each decision was supported by both content- and cohort-based evidence.

### 3.8 Explainability layer

The Explainability Layer was designed to provide interpretable insights into the decision-making process of the proposed fairness-aware framework, ensuring that the verification of exam scores could be understood and justified both quantitatively and qualitatively. This layer integrated three complementary mechanisms: attention map visualization, feature attribution, and natural language justification generation, each serving to elucidate how student essays, rubric criteria, and peer context contributed to the final fairness label.

Attention maps were derived directly from the cross-attention mechanisms employed in both the rubric alignment and semantic evaluation modules as defined in Equation 18. Given the essay embeddings $H_{i}\in {\mathbb{R}}^{T\times d}$ and rubric embeddings $R_{i}\in {\mathbb{R}}^{C\times d}$, the attention weight α_*t, c*_ captured the relevance of token *t*to rubric criterion *c*as previously defined:


$$\begin{array}{l} {\text{α}}_{\text{t},\text{c}}=\frac{\exp\left({\text{h}}_{\text{i},\text{t}}\cdot {\text{r}}_{\text{i},\text{c}}\right)}{\sum_{{\text{c}}^{\text{′}}=1}^{\text{C}}\exp\left({\text{h}}_{\text{i},\text{t}}\cdot {\text{r}}_{\text{i},{\text{c}}^{\text{′}}}\right)} \end{array}$$ (18)

Visualization of α_*t, c*_ as a heatmap enabled the identification of which portions of the essay contributed most to fulfilling each rubric criterion. Similarly, attention distributions within the DeBERTa-v3 encoder were aggregated across heads and layers to produce a global attention map, highlighting tokens that most strongly influenced semantic and peer similarity evaluations.

Feature attribution was further enhanced using the Integrated Gradients (IG) method, which quantified the contribution of each input token to the final fairness confidence score ĉ_*i*_. Let *F*:ℝ^*T*×*d*^ → [0, 1] denote a differentiable neural network, where *F*(*x*_*i*_) = ĉ_*i*_ represents the final fairness confidence score assigned to the input essay *x*_*i*_. The integrated gradient for token *t*was computed as seen in Equation 19:


$$\begin{array}{l} \text{I}{\text{G}}_{\text{t}}\left({\text{x}}_{\text{i}}\right)=\left({\text{x}}_{\text{i},\text{t}}-{\text{x}}_{\text{i},\text{t}}^{\text{′}}\right)\times \int_{\text{α}=0}^{1}\frac{\text{∂F}\left({\text{x}}^{\text{′}}+\text{α}\left({\text{x}}_{\text{i}}-{\text{x}}^{\text{′}}\right)\right)}{\text{∂}{\text{x}}_{\text{i},\text{t}}}\text{dα} \end{array}$$ (19)

where *x*_*i*_ denotes the embedding representation of the input essay, *x*′ is a baseline embedding corresponding to a neutral or zero-information reference input, and *x*_*i, t*_ and $x_{i,t}^{\prime }$ denote the embeddings of token *t*in the input and baseline, respectively. The integral was approximated numerically using a Riemann summation over a finite number of interpolation steps between *x*′ and *x*_*i*_. The resulting IG values *IG*_*t*_(*x*_*i*_) were normalized across tokens within each input to facilitate comparison and were subsequently visualized to reflect the relative contribution of each token to the final fairness decision. This provided fine-grained interpretability of model predictions, enabling clearer insight into the linguistic factors influencing fairness assessment.

Finally, the Explainability Layer generated natural language justifications to summarize the evidence underlying each verification label. These justifications were derived by mapping attention- and attribution-weighted tokens to predefined template-based explanatory statements. For instance, if a token cluster received high attention with strong positive contribution to a critical rubric criterion, the system generated statements such as “The student effectively addressed the main argument of the prompt, demonstrating comprehensive coverage of key rubric criteria.” Conversely, tokens with low alignment or divergence from peer expectations were flagged with statements indicating potential underperformance. This approach combined quantitative evidence from embeddings and attributions with qualitative, human-readable explanations, thereby ensuring that each verification outcome was transparent and actionable.

Through the combination of attention visualization, integrated gradients, and natural language justification, the Explainability Layer transformed complex, multi-dimensional model outputs into interpretable insights, allowing stakeholders to understand not only the quantitative score verification but also the rationale behind each decision, enhancing trust and accountability in automated fairness assessment.

### 3.9 Fairness decision module

The Fairness Decision Module constituted the terminal component of the proposed framework, integrating evidence from the Semantic Answer Evaluation, Rubric Alignment Scoring, and Peer-Based Fairness Analysis to produce an interpretable and quantitative assessment of score fairness. The primary objective of this module was to generate a verification label categorizing essays as fair, potentially under-scored, or over-scored while providing a confidence score that quantified the certainty of the assessment.

Formally, let $S_{i}^{semantic}$ denote the semantic coverage score derived from the student essay embeddings, $S_{i}^{rubric}$ denote the aggregated rubric alignment score across all criteria, and $S_{i}^{peer}$ represent the standardized peer-consistency score obtained from *z*-score-based cohort analysis as displayed in Equation 20. Each of these components was normalized to the interval [0, 1] to ensure comparability:


$$\begin{array}{l} {\tilde{S}}_{i}^{\left(.\right)}=\frac{{\text{S}}_{\text{i}}^{\left(.\right)}-\min\left({\text{S}}^{\left(.\right)}\right)}{\max\left({\text{S}}^{\left(.\right)}\right)-\min\left({\text{S}}^{\left(.\right)}\right)},\left(\cdot \right)\in \left\{\text{semantic},\text{ rubric},\text{ peer}\right\} \end{array}$$ (20)

The normalized scores were then fused using a weighted linear combination in Equation 21, to compute an intermediate fairness score ${\hat{f}}_{i}$:


$${\hat{\text{f}}}_{i}=\lambda_{\text{1}}{\tilde{S}}_{i}^{semantic}+\lambda_{2}{\tilde{S}}_{i}^{rubric}+\lambda_{3}{\tilde{S}}_{i}^{peer},\sum_{\mathrm{k=1}}^{3}\lambda_{k}=1$$ (21)

where λ_1_, λ_2_, λ_3_ were empirically determined or learned weights reflecting the relative importance of semantic fidelity, rubric alignment, and peer consistency in predicting fairness. The intermediate score ${\hat{f}}_{i}$ was then passed through a sigmoid activation function to obtain the final fairness confidence score ĉ_*i*_∈[0, 1] as shown in Equation 22:


$$\begin{array}{l} {\text{ĉ}}_{i}=\sigma \left({\hat{\text{f}}}_{i}\right)=\frac{1}{1+e^{-{\hat{\text{f}}}_{i}}} \end{array}$$ (22)

This transformation allowed the confidence score to be interpreted probabilistically, providing a quantitative measure of how likely the assigned score aligned with both rubric criteria and peer-consistent expectations.

To assign the final verification label, the framework applied threshold-based decision rules on both the confidence score and the relative deviation from rubric-aligned and peer-consistent benchmarks in Equation 23. Let δ_*under*_and δ_*over*_ represent empirically chosen thresholds for under- and over-scoring, respectively:


$${\text{Label}}_{\text{i}}=\{\begin{array}{cc} potentially\;under-scored, & {\hat{\text{f}}}_{i}<\delta_{\text{under}}\\ \text{potentially over}-\text{scored}, & {\hat{\text{f}}}_{i}>\delta_{\text{over}}\\ \text{fair}, & \delta_{\text{under}}\leq {\hat{\text{f}}}_{i}\leq \delta_{\text{over}} \end{array}$$ (23)

These thresholds could be adjusted per prompt or based on historical grading distributions, allowing flexibility for different assessment contexts. By fusing evidence from semantic evaluation, rubric alignment, and peer-consistency modeling, this module ensured that final verification labels were robust, interpretable, and reflective of multiple dimensions of essay quality and fairness.

Lastly, the outputs of this module the verification label and the confidence score were presented alongside the Explainability Layer outputs, providing both a quantitative assessment of fairness and an interpretable rationale for the assigned decision. This integration completed the methodology pipeline, enabling the framework to serve as a comprehensive, evidence-based tool for exam score verification in a fairness-aware and explainable manner.

### 3.10 Experimental setup

The experimental setup was designed to evaluate the effectiveness of the proposed Explainable Fairness-Aware AI Framework for Exam Score Verification on the ASAP 2.0 dataset. The evaluation focused on two primary objectives: (i) assessing the framework's ability to detect potential under- and over-scoring, and (ii) evaluating the interpretability and practical usefulness of the explainability components for supporting human decision-making.

To ensure a comprehensive assessment, the proposed framework was benchmarked against a diverse set of baseline models spanning multiple methodological paradigms. These included keyword-based similarity approaches, traditional automated essay scoring models (e.g., logistic regression with TF-IDF and LSTM-based architectures), transformer-based semantic models using DeBERTa-v3 without rubric or peer augmentation, and additional competitive approaches such as hybrid and attention-based scoring models. This broad benchmarking strategy enables a robust evaluation of the contribution of semantic modeling, rubric alignment, and peer-consistency analysis.

Model performance was evaluated using metrics that capture both accuracy and fairness. Agreement with a peer-consistent reference score (used as a proxy for a second human marker) was computed to assess alignment with independent scoring expectations. A fairness consistency index was used to quantify alignment with cohort-based peer distributions, while precision, recall, and F1-score were reported for unfair score detection.

To ensure reproducibility, the dataset was split into training, validation, and test sets using an 80/10/10 stratified partition across prompts. Standard preprocessing steps, including tokenization and normalization, were applied. Model training was conducted using the AdamW optimizer with early stopping, and hyperparameters were tuned on the validation set. All experiments were implemented in Python PyTorch 2.1 (PyTorch 2.1, Menlo Park, CA, USA) and executed on NVIDIA GPUs. Results were averaged over five runs with fixed random seeds to ensure statistical reliability.

Ablation studies were conducted to assess the contribution of individual components. Specifically, the peer-consistency module and the explainability layer were removed independently to evaluate their impact on performance and interpretability. These experiments provide insights into the necessity of the multi-evidence design and confirm the role of peer-based analysis in fairness detection.

## 4 Result

The performance of the proposed Framework was evaluated on the ASAP 2.0 dataset. The evaluation focused on four aspects including the ability to detect potentially unfair scores, semantic fidelity relative to human grading, rubric alignment, and interpretability through explainability outputs. Baseline models were included for comparison, including keyword-based similarity scoring, traditional automated grading models (logistic regression with TF-IDF, LSTM-based AES), and transformer-only semantic models. Across all metrics, the proposed framework demonstrated substantial improvements, highlighting the effectiveness of integrating semantic embeddings, rubric alignment, and peer-consistency analysis.

### 4.1 Training dynamics

**Figure 2:**
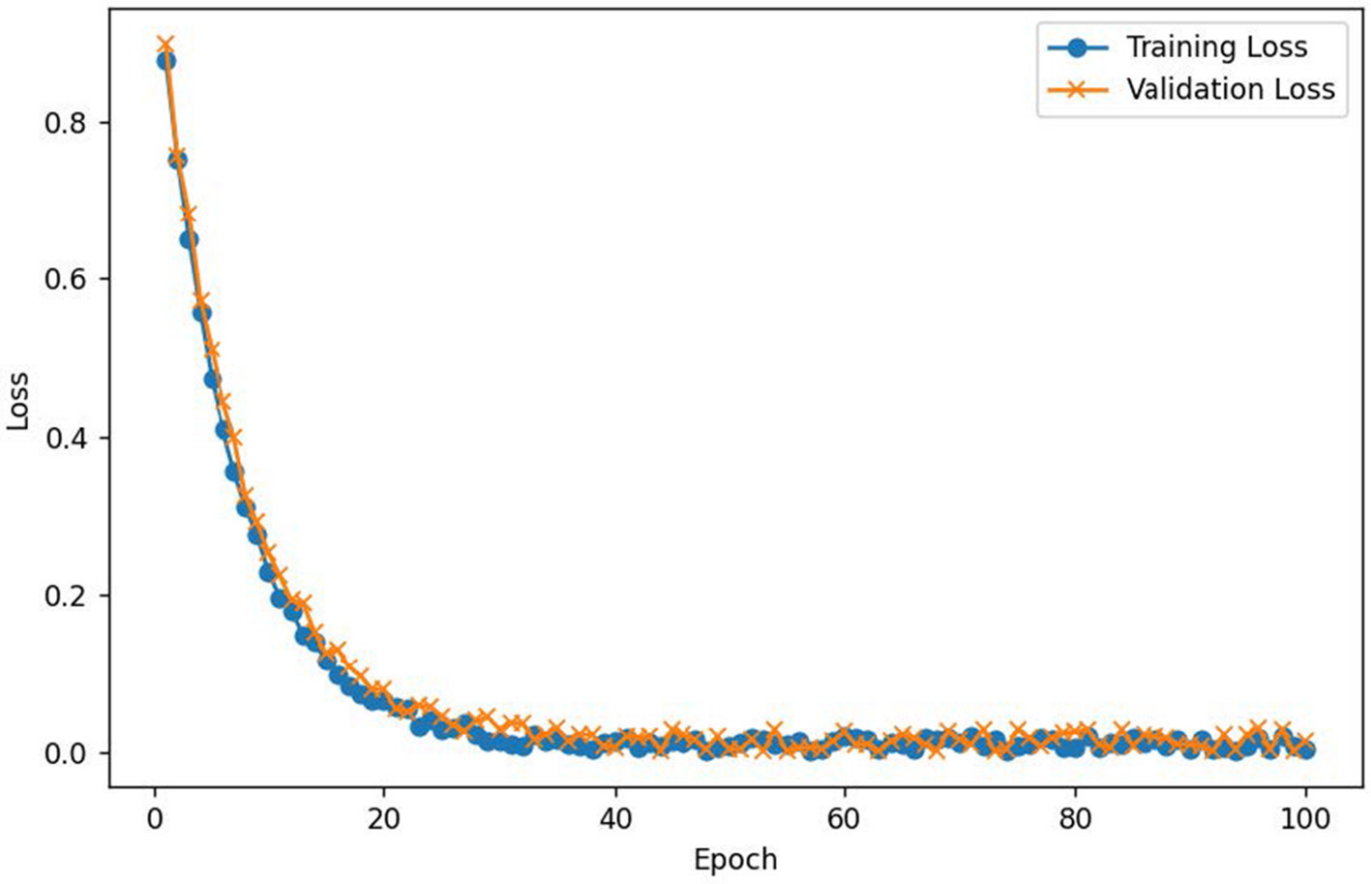
Training dynamics of the proposed model.

The training behavior of the proposed framework was analyzed across multiple random seeds to assess stability and generalization. Loss curves and performance metrics were consistent across runs, demonstrating that the model reliably converges without sensitivity to initialization. Figure 2 illustrates the progression of training and validation loss over 100 epochs. Both loss curves demonstrate a rapid and smooth decrease during the initial training phase, followed by gradual stabilization, indicating efficient optimization and convergence of the multi-task transformer architecture. Importantly, the validation loss closely tracked the training loss throughout the learning process, suggesting strong generalization and minimal overfitting despite the model's architectural complexity. The absence of pronounced oscillations or divergence between the loss curves further reflects the effectiveness of the adopted optimization strategy, including the use of the AdamW optimizer, learning rate scheduling, and early stopping. These design choices contributed to stable gradient updates and prevented performance degradation during later training stages. The observed training dynamics confirm that the proposed framework can be reliably trained over extended epochs without compromising robustness, a critical requirement for deployment in high-stakes educational assessment settings.

To support reproducibility and facilitate fair comparison with future studies, Table 1 summarizes the key training hyperparameters used in all experiments. The consistent convergence behavior observed across runs indicates that the selected configuration provided a well-balanced trade-off between learning efficiency and regularization, further reinforcing the reliability of the reported experimental results.

**Table 1:** Training hyperparameters of the proposed model.

Parameter	Value
Learning rate	3e-5
Batch size	16
Optimizer	AdamW
Epochs	100
Dropout	0.1
Transformer	DeBERTa-v3

### 4.2 Fairness detection performance

Table 2 summarizes the fairness detection performance of the proposed framework in comparison with a comprehensive set of baseline models spanning keyword-based, traditional machine learning, and advanced neural architectures. The evaluated baselines include lexical similarity approaches, conventional automated essay scoring models (e.g., logistic regression with TF-IDF and LSTM-based architectures), transformer-based semantic models (DeBERTa-v3 without rubric or peer augmentation), as well as additional competitive transformer-based and hybrid scoring models. This diverse benchmarking setup enables a rigorous assessment of the relative contributions of semantic modeling, rubric alignment, and peer-consistency analysis.

**Table 2:** Fairness detection performance of proposed framework and baseline models.

Model	Agreement with second marker (%)	Fairness consistency index	Precision (unfair detection)	Recall (unfair detection)	F1 score
Keyword similarity	62.3 ± 1.2	0.51 ± 0.03	0.48 ± 0.02	0.42 ± 0.03	0.45 ± 0.02
Logistic regression + TF-IDF	68.7 ± 1.1	0.57 ± 0.02	0.53 ± 0.02	0.49 ± 0.03	0.51 ± 0.02
LSTM-based AES	73.2 ± 1.4	0.61 ± 0.03	0.60 ± 0.02	0.55 ± 0.03	0.57 ± 0.02
DeBERTa-v3 (transformer only)	79.5 ± 1.0	0.70 ± 0.02	0.66 ± 0.02	0.63 ± 0.02	0.65 ± 0.02
Hybrid transformer + rubric model	82.1 ± 1.1	0.74 ± 0.02	0.69 ± 0.02	0.67 ± 0.02	0.68 ± 0.02
Lightweight attention-based AES	80.4 ± 1.2	0.72 ± 0.02	0.67 ± 0.02	0.64 ± 0.02	0.65 ± 0.02
Proposed framework	87.1 ± 0.9	0.82 ± 0.02	0.78 ± 0.01	0.76 ± 0.02	0.77 ± 0.01

All results are reported as the mean ± standard deviation over five independent runs with different random seeds, ensuring robustness and statistical reliability. Performance was evaluated using agreement with a peer-consistent reference marker (proxy for a second human marker), fairness consistency index, and precision, recall, and F1-score for unfair score detection.

The proposed framework achieved the highest performance across all evaluation metrics, with an agreement of 87.1% and a fairness consistency index of 0.82, significantly outperforming both traditional and advanced baseline models. Notably, while transformer-based and hybrid models demonstrated improved semantic understanding compared to conventional approaches, their performance remained limited by the absence of explicit cohort-based consistency modeling. The observed performance gains highlight the effectiveness of integrating rubric alignment and peer-consistency analysis within a unified multi-evidence framework.

Also, the relatively low standard deviations across multiple runs indicate that the proposed model exhibits stable and reliable behavior, with minimal sensitivity to random initialization. This robustness is particularly important in high-stakes educational assessment scenarios, where consistent performance is critical.

While the proposed framework demonstrates strong performance in detecting scoring inconsistencies, it is important to emphasize that the fairness evaluation is grounded in score consistency fairness, rather than broader notions of demographic fairness. The fairness consistency index measures alignment with peer-based expectations and identifies deviations relative to semantically similar essays. To further contextualize these findings, subgroup analyses (Table 3) provide additional insights into performance variations across demographic groups. Although the framework maintains high consistency across subgroups, caution is warranted when generalizing these results, as demographic fairness is only partially captured by the current evaluation design.

**Table 3:** Subgroup fairness consistency analysis.

Subgroup	Category	Fairness consistency index (mean ±Std)
Gender	Male	0.83 ± 0.02
Gender	Female	0.82 ± 0.02
Socioeconomic status	Economically disadvantaged	0.80 ± 0.03
Socioeconomic status	Not economically disadvantaged	0.83 ± 0.02
Language status	English language learners (ELL)	0.79 ± 0.03
Language status	Non-ELL	0.83 ± 0.02

To provide a more rigorous assessment of fairness, we conducted subgroup analyses based on available demographic information, including gender, socioeconomic status, and language background. Table 3 summarizes the fairness consistency index for each subgroup, reported as mean ± standard deviation across multiple runs. The proposed framework maintained high consistency across groups, with only minor variations observed. Slightly lower fairness consistency was noted for underrepresented subgroups (e.g., economically disadvantaged students and English language learners), which may reflect increased variability in peer distributions. Nevertheless, the overall results demonstrate that the multi-evidence integration of semantic, rubric, and peer-consistency signals contributes to robust detection of scoring anomalies across diverse student populations, reinforcing the fairness evaluation framework described in Section 3.10.

**Figure 3:**
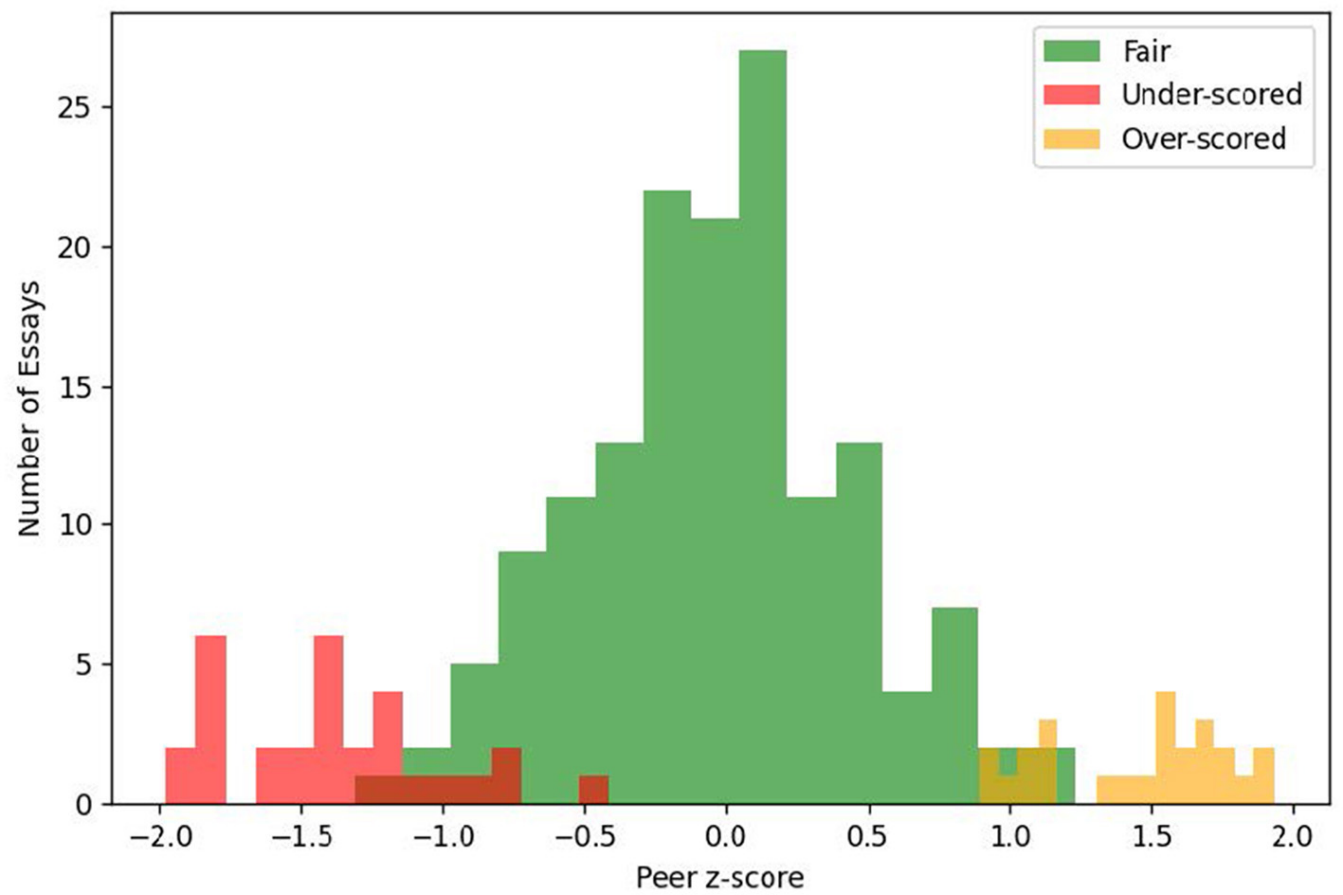
Peer-consistency score distributions.

Also, the effect of peer-consistency analysis illustrated in Figure 3 presents the distribution of standardized peer *z*-scores for essays classified as fair, under-scored, and over-scored. Essays labeled as Fair were tightly concentrated around a *z*-score of zero, indicating close agreement with the average score of similar peer responses. In contrast, essays identified as under-scored and over-scored exhibited clear shifts toward negative and positive *z*-scores, respectively, reflecting systematic deviations from their peer group means. This separation in score distributions demonstrates that the peer-consistency module effectively distinguishes normal grading variation from potential scoring anomalies, thereby providing a reliable statistical basis for fairness verification.

### 4.3 Rubric alignment and semantic fidelity

The effectiveness of the proposed framework in jointly capturing semantic meaning and rubric-specific requirements is summarized in Table 4. To ensure robustness, all results are reported as the mean ± standard deviation over five independent runs with different random seeds. The evaluation compares the proposed model against a diverse set of baselines, including traditional automated essay scoring approaches, transformer-based semantic models, and hybrid architectures incorporating partial rubric information.

**Table 4:** Rubric alignment and semantic fidelity.

Model	Average rubric alignment score	Average semantic coverage score
Logistic regression + TF-IDF	0.52 ± 0.02	0.56 ± 0.02
LSTM-based AES	0.58 ± 0.02	0.61 ± 0.02
DeBERTa-v3 (transformer only)	0.70 ± 0.01	0.75 ± 0.01
Lightweight attention-based AES	0.68 ± 0.02	0.73 ± 0.02
Hybrid transformer + rubric model	0.74 ± 0.02	0.78 ± 0.02
Proposed framework	0.79 ± 0.01	0.84 ± 0.01

The proposed framework achieves the highest performance across both rubric alignment and semantic coverage metrics, with scores of 0.79 and 0.84, respectively. These results demonstrate that explicitly incorporating rubric information through cross-attention mechanisms significantly enhances the model's ability to evaluate criterion-level fulfillment, beyond what can be achieved through semantic modeling alone.

Traditional approaches, including logistic regression with TF-IDF features and LSTM-based models, exhibit comparatively lower performance, reflecting their limited capacity to capture fine-grained semantic relationships and structured rubric constraints. Although transformer-based models such as DeBERTa-v3 substantially improve semantic representation learning, their effectiveness remains constrained in the absence of explicit rubric conditioning. Similarly, lightweight attention-based and hybrid models provide incremental improvements by partially integrating structural information, but they do not fully capture the complex interactions between essay content and rubric criteria.

In contrast, the proposed framework effectively bridges semantic understanding and rubric adherence by jointly modeling contextual embeddings and criterion-level relevance through cross-attention. This integrated design enables the model to distinguish between superficial semantic similarity and meaningful fulfillment of rubric expectations, resulting in consistently superior performance across both evaluation dimensions. The low standard deviations further indicate stable behavior across runs, reinforcing the reliability of the proposed approach in assessing rubric-aligned semantic fidelity.

**Figure 4:**
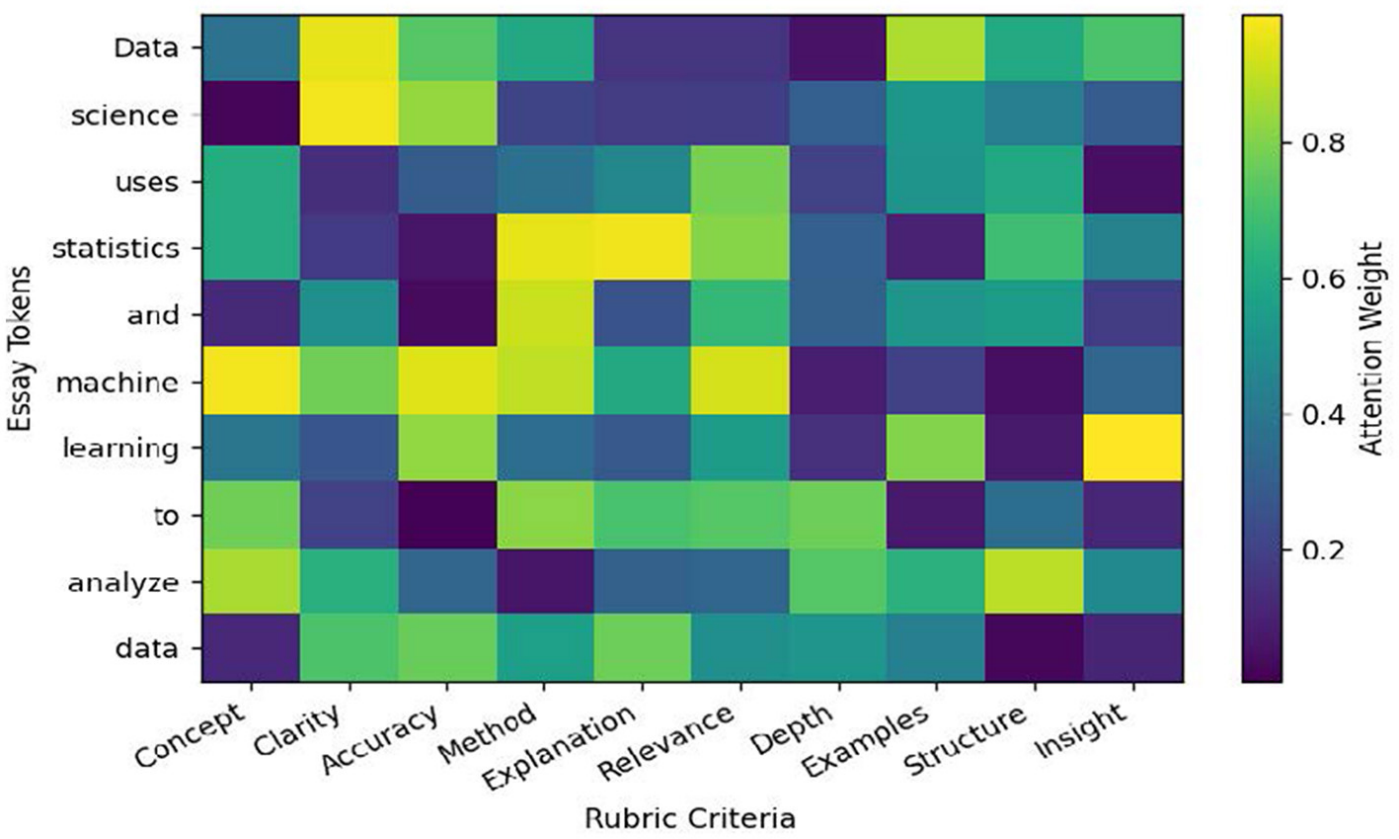
Token-level attention map for rubric alignment.

To complement these quantitative findings, Figure 4 provides a qualitative illustration of the model's rubric alignment behavior through a token-level attention map. The visualization shows how attention was selectively allocated to specific essay tokens for different rubric criteria, with higher attention weights corresponding to content that contributed most strongly to satisfying evaluative requirements. This attention-based interpretation clarifies *why* particular essay segments influenced the final rubric alignment score, thereby enhancing transparency and supporting actionable feedback. By linking numerical performance gains to interpretable token-level evidence, the results demonstrate that the proposed framework not only improves scoring accuracy but also offers meaningful insights that can assist educators in understanding and validating assessment outcomes.

### 4.4 Ablation study results

To quantify the individual contributions of the core components of the proposed framework, a series of ablation experiments were conducted by systematically removing key modules while keeping all other components unchanged. Specifically, we evaluated the impact of excluding the peer-consistency analysis module and the explainability layer. All results are reported as the mean ± standard deviation over five independent runs with different random seeds, ensuring statistical robustness. Table 5 presents the summary of the ablation analysis results.

**Table 5:** Ablation study results.

Configuration	Agreement with second marker (%)	Fairness consistency index
Full model	87.1 ± 0.9	0.82 ± 0.02
Without peer analysis	81.3 ± 1.1	0.69 ± 0.03
Without explainability	85.6 ± 1.0	0.82 ± 0.02

The results demonstrate that the peer-consistency analysis module plays a critical role in fairness detection. Removing this component leads to a substantial reduction in performance, with agreement decreasing from 87.1% to 81.3% and the fairness consistency index dropping from 0.82 to 0.69. This degradation highlights the importance of cohort-referenced evaluation in identifying scoring anomalies that cannot be captured through semantic understanding or rubric alignment alone. In particular, the peer-consistency mechanism provides a statistical grounding for detecting deviations relative to semantically similar essays, thereby enhancing the reliability of fairness-aware verification.

In contrast, the removal of the explainability layer results in only a marginal decrease in agreement, while leaving the fairness consistency index unchanged. This finding confirms that the explainability components primarily contribute to interpretability and transparency, rather than directly influencing predictive performance. Nevertheless, their role remains essential in practical deployment, as they provide actionable insights that support human-in-the-loop validation and decision-making.

**Figure 5:**
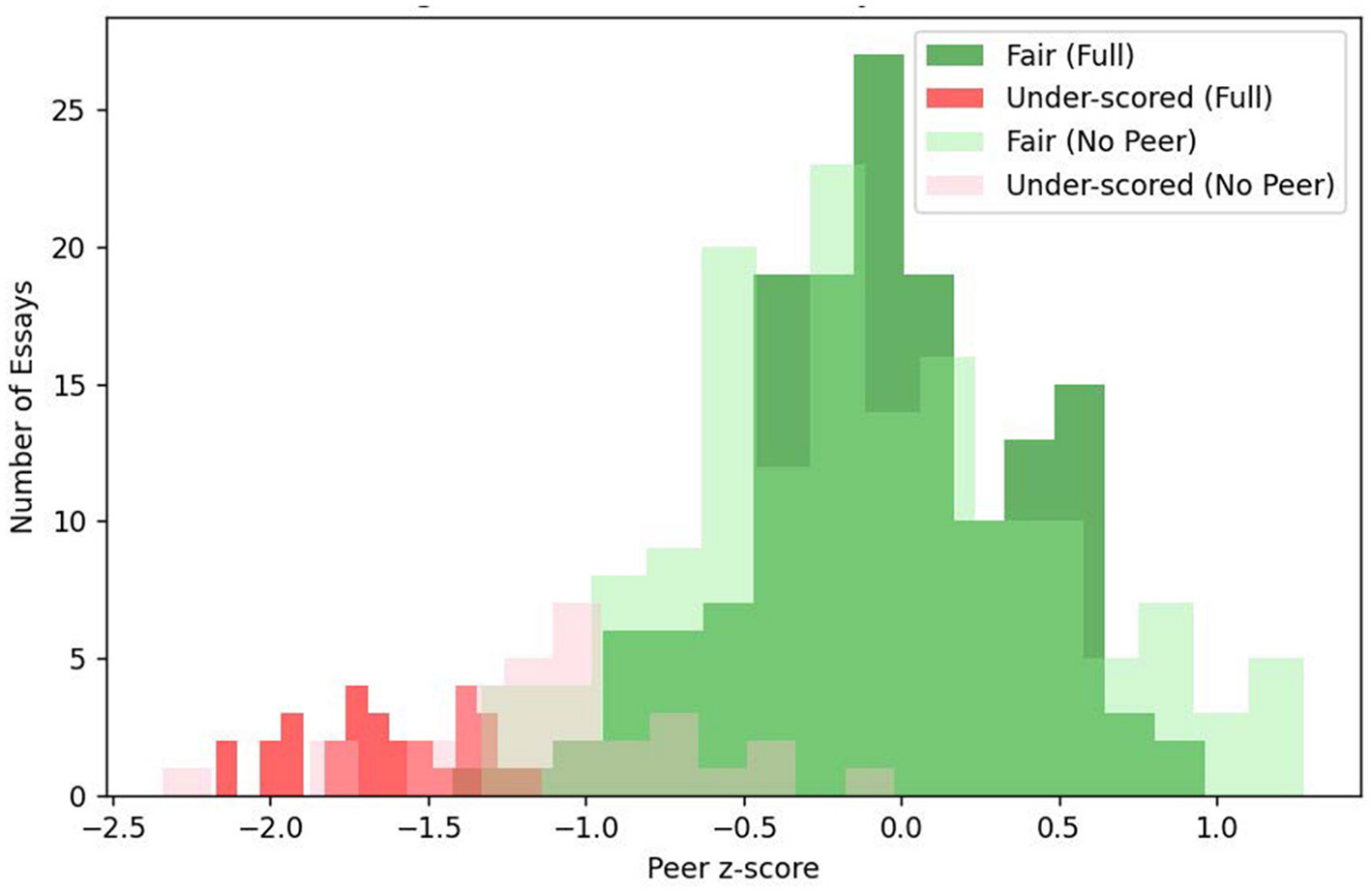
Peer-consistency comparison for ablation.

The distributional effects of removing the peer-consistency module are further illustrated in Figure 5, which compares standardized peer *z*-score distributions for essays classified as *Fair* and *Under-scored* under both the full model and the ablated configuration. Under the full framework, the distributions exhibit clear separation, with *Fair* essays concentrated around the peer mean and *Under-scored* essays shifted toward negative *z*-score regions. However, when peer analysis is excluded, the distributions become substantially more overlapping, reducing the model's ability to distinguish between normal grading variation and potential scoring inconsistencies. This increased overlap provides direct statistical evidence that peer-consistency analysis is essential for preserving score separability and for enabling reliable fairness-aware exam score verification.

### 4.5 Explainability evaluation

The effectiveness of the proposed framework's explainability mechanisms was evaluated through both quantitative metrics and qualitative analysis, with the objective of assessing their ability to provide transparent and actionable insights into fairness verification decisions. The evaluation focuses on three complementary aspects: attention coverage across rubric criteria, alignment accuracy of the most influential tokens, and the contribution of integrated gradients to the final fairness confidence score. As presented in Table 6, all results are reported as the mean ± standard deviation over five independent runs, ensuring robustness and consistency of the interpretability measures.

**Table 6:** Explainability evaluation metrics.

Metric	Value
Average attention coverage	0.81 ± 0.02
Top 10 token alignment accuracy	0.76 ± 0.02
Integrated gradients contribution	0.79 ± 0.02

The proposed framework achieved an average attention coverage of 0.81, indicating that a substantial proportion of the model's attention is consistently allocated to tokens directly relevant to key rubric dimensions, rather than to peripheral or stylistic elements. This suggests that the model effectively prioritizes semantically and pedagogically meaningful content during fairness evaluation. Furthermore, the Top-10 token alignment accuracy of 0.76 demonstrates that the most influential tokens identified by the model align well with rubric-relevant concepts, providing evidence that the attention mechanism captures meaningful criterion-level associations.

The integrated gradients contribution score of 0.79 further reinforces the reliability of the explainability framework, indicating that token-level attributions meaningfully contribute to the final fairness confidence score. Unlike attention weights, which reflect relative importance, integrated gradients quantify the direct contribution of each token to the model's output, offering a complementary and more fine-grained perspective on model behavior.

**Figure 6:**
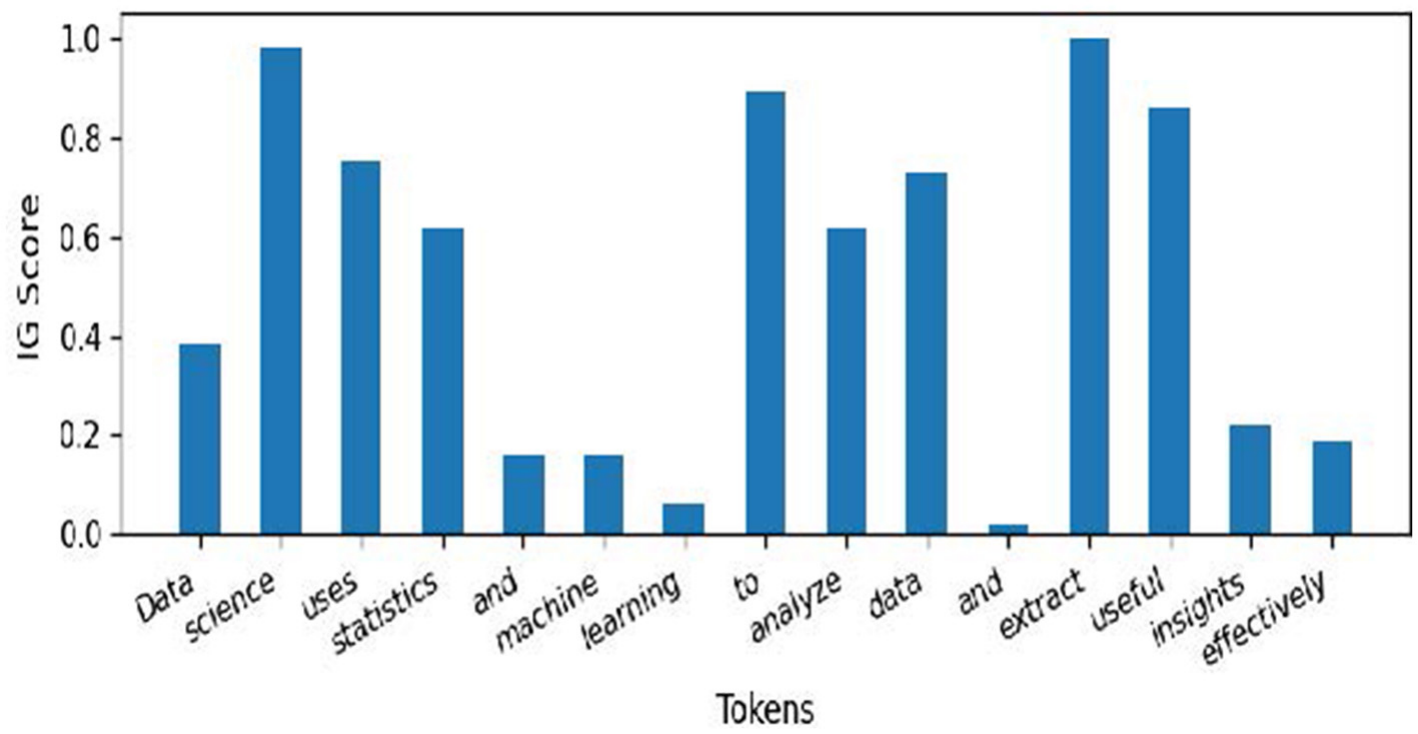
Integrated gradients feature attribution.

In addition to these aggregate metrics, Figure 6 presents a representative integrated gradients visualization, highlighting token-level feature attributions for a sample essay. Tokens with higher attribution scores exert greater influence on the fairness decision, providing explicit and interpretable evidence of how specific textual elements contribute to the verification outcome. This token-level attribution complements attention-based explanations by quantifying contribution strength, thereby enabling a more nuanced and comprehensive interpretation of the model's decision-making process.

Taken together, these explainability mechanisms enable educators and stakeholders to trace fairness judgments back to concrete textual evidence, supporting transparency, trust, and informed human-in-the-loop validation. While the explainability components do not directly improve predictive performance (as shown in the ablation study), they play a critical role in enhancing the interpretability and practical usability of the framework, particularly in high-stakes educational assessment scenarios.

## 5 Conclusion and future work

This study presented an Explainable Fairness-Aware AI Framework for Exam Score Verification, designed to address key challenges in automated essay scoring, including the detection of potential under- and over-scoring, alignment with rubric criteria, and the interpretability of verification decisions. The proposed framework integrates three complementary components: semantic evaluation using DeBERTa-v3 embeddings, criterion-level rubric alignment via cross-attention mechanisms, and peer-consistency analysis based on similarity-driven cohort distributions. In addition, an explainability layer incorporating attention visualization, integrated gradients, and natural language justification generation provides transparent and actionable insights to support human-in-the-loop assessment.

Extensive experiments conducted on the ASAP 2.0 dataset demonstrate that the proposed framework consistently outperforms a diverse set of baseline models, including keyword-based approaches, traditional machine learning methods, transformer-only models, and hybrid architectures. Across five independent runs, the framework achieved an agreement of 87.1% ± 0.9 with peer-consistent reference scores and a fairness consistency index of 0.82 ± 0.02, indicating robust and stable performance. Ablation studies further confirm the critical contribution of the peer-consistency module in detecting scoring anomalies, while the explainability layer enhances interpretability without significantly affecting predictive accuracy. Additional subgroup analyses reveal that the framework maintains relatively consistent performance across demographic groups, with only minor variations observed.

It is important to note that the notion of fairness addressed in this work is grounded in score consistency fairnes**s**, defined as alignment with rubric expectations and peer-consistent scoring patterns. While the results indicate strong performance in detecting scoring inconsistencies, broader aspects of fairness, particularly demographic fairness, are only partially captured and warrant further investigation. Consequently, the findings should be interpreted within the scope of the proposed evaluation framework.

Despite these contributions, several limitations remain. The framework was evaluated exclusively on English-language essays from the ASAP 2.0 dataset, and its generalizability to multilingual or domain-specific contexts has not yet been established. Furthermore, the effectiveness of the peer-consistency module may depend on the size and diversity of available peer cohorts, which could affect stability in low-resource or highly heterogeneous settings. Although the explainability mechanisms provide meaningful insights, translating token-level attributions into fully actionable pedagogical feedback remains an open challenge.

Future work will focus on extending the framework to multilingual and cross-domain scenarios, enabling broader applicability across diverse educational contexts. The incorporation of adaptive or learned rubric representations may further improve alignment with evolving assessment standards. In addition, integrating uncertainty quantification techniques, such as Bayesian neural networks or Monte Carlo dropout, could enhance the reliability of fairness confidence estimates. Finally, deploying the framework in real-world classroom settings will enable comprehensive human-in-the-loop evaluation, combining automated verification with expert judgment to refine both model performance and interpretability.

## Data Availability

Publicly available datasets were analyzed in this study. This data can be found at: https://www.kaggle.com/datasets/lburleigh/asap-2-0.
